# Examination of equine glandular stomach lesions for bacteria, including *Helicobacter spp *by fluorescence *in situ *hybridisation

**DOI:** 10.1186/1471-2180-10-84

**Published:** 2010-03-19

**Authors:** Louise Husted, Tim K Jensen, Susanne N Olsen, Lars Mølbak

**Affiliations:** 1 Department of Large Animal Sciences, Faculty of Life Sciences, University of Copenhagen, Hoejbakkegaard Allé 5, 2630 Taastrup, Denmark; 2 Danish Veterinary Institute, Technical University of Denmark, Bülowsvej 27, 1790 Copenhagen V, Denmark

## Abstract

**Background:**

The equine glandular stomach is commonly affected by erosion and ulceration. The aim of this study was to assess whether bacteria, including Helicobacter, could be involved in the aetiology of gastric glandular lesions seen in horses.

**Results:**

Stomach lesions, as well as normal appearing mucosa were obtained from horses slaughtered for human consumption. All samples were tested for urease activity using the Pyloritek^® ^assay, while mucosal bacterial content was evaluated using Fluorescence *In Situ *Hybridisation. In selected sub samples, bacteria characterisation was pursued further by cloning and sequencing. Mucosal lesions were found in 36/63 stomachs and included hyperplastic rugae, polypoid structures and focal erosions. None of the samples were tested positive for urease activity or for FISH using the Helicobacter genus specific probe. In samples of lesions, as well as normal samples, clones with 99% similarities to *Lactobacillus salivarius *and *Sarcina ventriculi *were found. *Escherichia *like bacterium clones and Enterococcus clones were demonstrated in one focal erosion. Based on a phylogenetic tree these clones had 100% similarity to *Escherichia fergusonii and Enterococcus faecium*. The Enterococcus were found colonising the mucosal surface, while *E. fergusonii *organisms were also demonstrated intraepithelial.

**Conclusion:**

Gastric Helicobacter spp. could not be verified as being involved in lesions of the glandular stomach of the horse. Since *E. fergusonii *has been described as an emerging pathogen in both humans and animals, the finding of this bacterium in gastric erosion warrants further clarification to whether gastric infection with this type bacterium is important for horses.

## Background

In horses, lesions of the non-glandular part of the stomach are highly prevalent and seem to be caused by excessive acid exposure [1], but little has been described regarding lesions in the glandular part. Lesions located in the glandular region were demonstrated in 58% of 162 hospitalized horses [2] and in 47% of 345 racehorses [3] and while the cause of these have not received much attention, acid exposure does not seem to be the primary factor, as no correlation between lesions of the two regions of the stomach has been found [3].

Gastric bacteria as the cause for glandular stomach lesions have been suggested for many animal species and in humans these constitute a major verified risk factor. Of the gastric organisms found, *Helicobacter pylori *has been described the most due to its pathogenic potential of inducing chronic gastritis, ulcers, adenocarcinomas and mucosa associated lymphoid tissue (MALT) lymphoma in humans [4-6]. Bacteria of this genus have also been found in gastric tissue samples from animals including dogs, pigs, sheep and cattle [7-10].

In the horse, contradictory evidence exits as to whether bacteria that specifically can cause gastric lesions occur. A few studies have indicated that gastric *Helicobacter spp*. are present in normal appearing mucosa by using PCR and immunochemistry [11,12], while others have found no evidence of a connection between the presence of lesions and bacteria [13]. As gastric bacterial species have been confirmed or suggested as part of the pathogenesis of certain types of gastric pathology in humans and other animal species, the aim of this study was to assess if bacteria could be involved in the pathology observed in the equine glandular stomach. A main focus was to provide more evidence regarding the presence and localisation of bacteria in general at the mucosa level of the equine glandular stomach. Special emphasis was put on obtaining information regarding the presence and involvement of any *Helicobacter species *in the mucosal lesions. The Fluorescence *In situ *hybridisation (FISH) technique was used for this purpose which allows the use of rRNA-targeted probes for both the total bacterial population and defined genus/species. This approach permits the determination of bacterial morphology, abundance, location in the tissues, and even indications on growth rates and physiological activities [14].

## Results

Gross glandular lesions were seen in 36 of the 63 stomachs examined (57.1%). The majority of lesions were seen in the antrum region (91.7%). In six stomachs, lesions were additionally or exclusively seen in the cardia or corpus region. No lesions were found in the duodenum.

The lesions were classified in three groups as: Polypous (2 stomachs with polypoid masses located in both the cardia and the antrum with sizes between 1 and 5 centimetres in diameter), ii: Hyperplastic rugae lesions (13 stomachs) or iii: Hyperaemic, erosive or ulcerative lesions, which were seen in 21 stomachs.

The hyperplastic rugae were all seen in the antrum and ranged from having intense hyperemia with exudate to rugae with normally appearing mucosal surface. Gross thickening of the antrum rugae was caused primarily by hyperplasia of the gastric foveolae compared to the respective normal samples. The remaining lesions were all found to be small solitary lesions of no more than approximately 1 × 2 cm in size. Focal areas of erosive gastritis was the most common findings of these type lesions and characterised as sloughing of the superficial cells of the luminal epithelium with a concurrent fibrinopurulent exudate, luminal cellular debris and a predominantly mononuclear cell infiltrate of the lamina propria. Deeper erosions found in 9 stomachs eroded both the region of the gastric pits and parts of the glands, which was observed with gastritis only of the immediate tissues. One true ulcer was found extending the full thickness of the lamina propria, exposing the lamina muscularis to the lumen. A maximum of two lesions were found in each of these stomachs.

### Helicobacter and Urease activity test

Using the genus Helicobacter specific probe no positive signals were found in any of the 79 tissue samples (36 paired samples and 7 controls). In agreement with these results of the FISH, none of the samples tested positive for urease activity either. Internal controls of all urease tests were found positive as indication of a functional test kit.

### Bacteria in general

In general, only few bacteria were observed related to the mucosal surface in both the injured as well as in the healthy stomach samples. Overall, four morphological different types of bacterial cells could be visualized with the Eubacteria probe: 1) small, short (0.2-0.5 μm) coccoid rods, 2) distinct rods (1 × 3 μm), 3) long chained rods (up to 60 μm) or 4) large (2-3 μm diameter) coccoid bacteria clearly dividing in pairs. Typically when present, bacteria were observed in clusters associated with feed particles or located close to the mucosal surface

**Figure 1:**
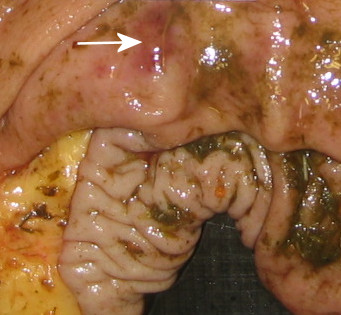
**Focal erosive lesion (white arrow) demonstrating bacterial gastritis at histological evaluation**. Lesion was approximately 2 × 2 cm and located in the antrum near the pyloric entrance.

**Figure 2:**
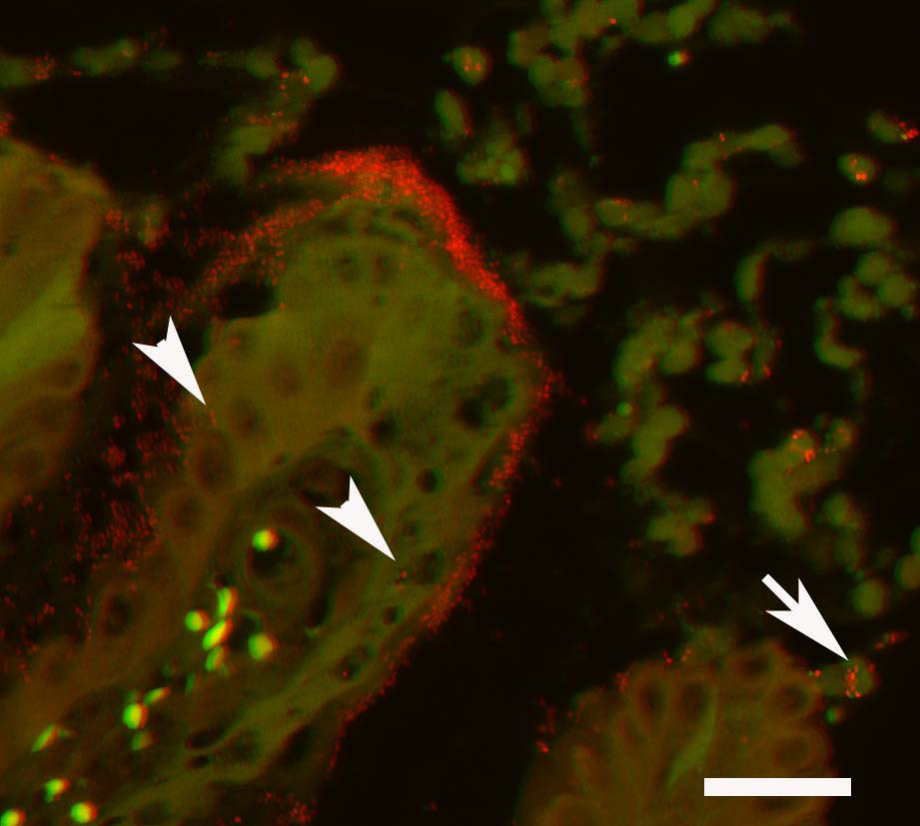
**Gastric mucosa with erosive gastritis associated with bacteria**. The mucosal surface and adjacent cellular debris is severely colonised by bacteria (red). A few bacteria are seen intracellular in the intact epithelium (arrowhead) as well as within degenerated and necrotic epithelial cells (arrow). In addition, bacteria are found within granulocytes. Fluorescent in situ hybridisation with the probe targeting Bacteria, filter set 43, bar = 25 μm.

Evidence of bacterial gastritis was found in one stomach lesion grossly characterised as a solitary erosion, 1 × 2 cm in size, the centre being hyperaemic and surrounded by a proliferative epithelial rim (Fig. 1). Microscopically, focal erosion of the mucosa with oozing of erythrocytes and leukocytes, mainly of neutrophilic origin, was seen. The exudates were additionally seen in the gastric pits. A cellular inflammatory reaction with mononuclear cells was seen extending as deep as into the lamina muscularis. The surface of the inflamed mucosa and the gastric pits were found heavily colonised by coccoid to short rods applying the probe for general bacteria (Fig. 2). The short rods were especially observed infiltrating the erosion. They were also observed intracellular in epithelial cells, as well as within neutrophilic granulocytes. The bacterial colonisation of the stomach was restricted to the lesion as no bacteria were seen in the corresponding healthy mucosa sample.

### Cloning and sequencing

Based on the morphology and intensity of bacteria demonstrated using FISH, subsamples of the C/c samples were selected for cloning and sequencing of representing samples including the one with bacterial gastritis.

Of the chosen subsamples of stomachs demonstrating various bacteria morphologies, two different types of clones were found in normal appearing mucosa samples (c samples), one clone had 99% similarity to *Lactobacillus salivarius *JCM 1231 (AB370881) and the other type of clones had 99% similarity to *Sarcina ventriculi *DSM 316 (X76650).

**Figure 3:**
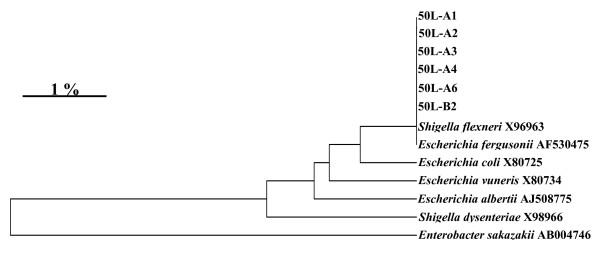
**A phylogenetic tree of 16S rRNA gene sequence similarity, showing the position of the six clones belonging to Gammaproteobacteria found in Horse 50L and the most closely related type strains belonging to the *Escherichia *genus**. The six clones (acc.no. GQ423062) had 100% similarity to *Shigella flexneri *and *E. fergusonii*. Enterobacter sakazakii (AB004746) was used as an outgroup. Sequence accession numbers are presented.

**Figure 4:**
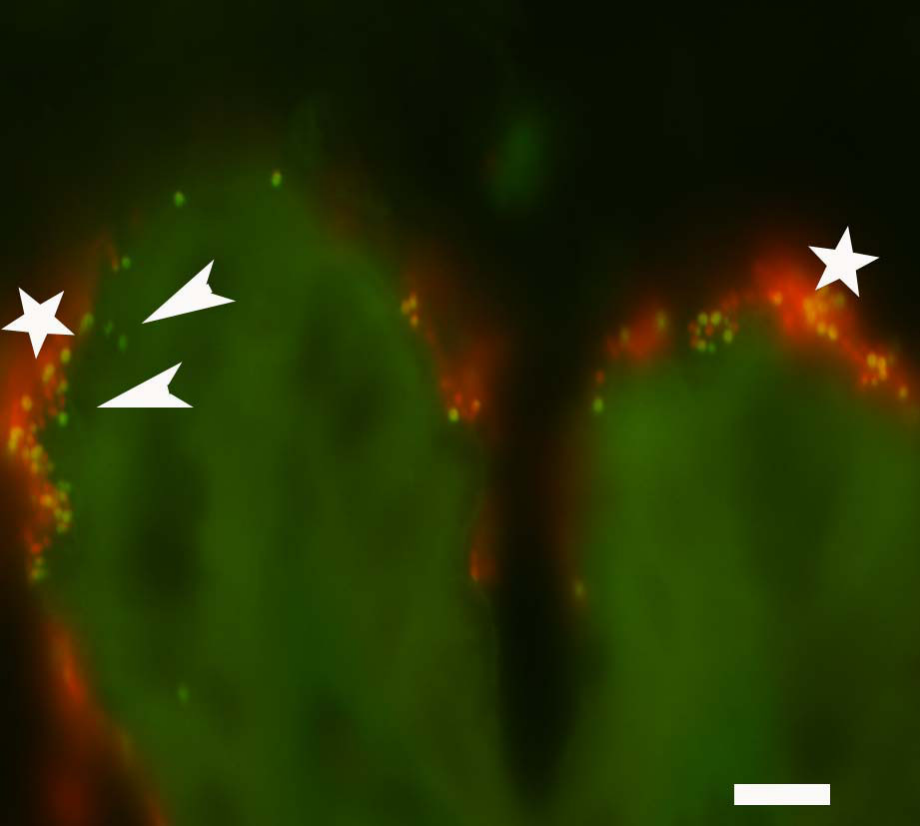
**Gastric mucosa of horse 50L with erosive gastritis associated with bacteria**. Applying a fluorescein labelled probe for Gammaproteobacteria and a Cy3 labelled probe for Enterococcus, an *E. coli *like organism (green) (arrowhead) was found intracellular within epithelial cells and on the epithelial surface whereas E. faecium (red) ('white star'(only colonised the epithelial surface. Filter set 43/38, bar = 10 μm.

**Figure 5:**
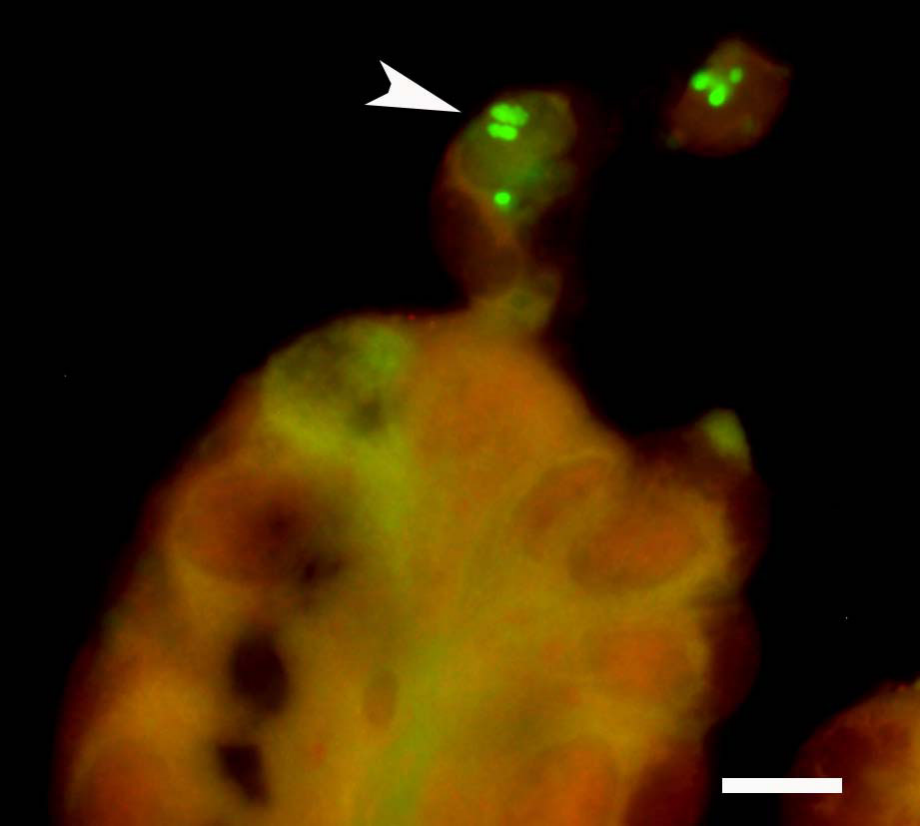
**Gastric mucosa of horse 50L with erosive gastritis associated with bacteria**. High magnification demonstrating *E. coli *like rods (green) within extruded epithelial cells. Fluorescent in situ hybridisation with the probe targeting Gammaproteobacteria, filter set 38, bar = 10 μm.

From the lesions (C samples), clones were also found with 99% similarity to *Lactobacillus salivarius *JCM 1231 (AF182725). From the mucosa with bacterial gastritis, four of ten clones matched 100% *Enterococcus faecium*, while the remaining six clones (obtained sequence deposited at GenBank with the accession no. GQ423062) belonged to an *Escherichia like *bacterium. A phylogenetic tree was constructed with the six *Escherichia *like clones from the lesion and all had 100% similarity to the type strains of both *E. fergusonii *and *Shigella flexneri *(fig 3). Applying a gamma proteobacteria specific probe the short rods infiltrating the epithelium, as well as found intracellular within neutrophilic granulocytes, were verified as the *Escherichia *like bacterium while *Enterococcus faecium *organisms were identified colonising the epithelial surface by the Enterococcus specific probe (Fig 4 and 5).

## Discussion

Previous studies involving the equine stomach have e.g. used PCR targeting the 16S rRNA gene of especially *Helicobacter *spp. [12]. The disadvantages using PCR are that the amount and location of the bacteria is not known and it is uncertain whether the bacteria are alive or even if the DNA is naked. Hence, it was decided that using the FISH technique would provide better and more information of the bacteria found in the glandular stomach of the horse, as these issues are overcome with this technique. This technique has been used previously to describe the spatial distribution of *Helicobacter *spp. in the gastrointestinal tract of dogs and in the stomach of healthy horses to demonstrate the microbiota of the normal appearing squamous and glandular mucosa [15,16]. To the best of our knowledge this is the first study using FISH to examine lesions of the glandular stomach.

In the present study one case of gastritis associated with bacterial colonisation was revealed. Especially the distribution of bacteria suggested a connection with the pathology observed. The amount of bacteria was markedly increased around the lesion and were tightly adhered to the epithelial cells, with the bacteria extending into the crypts and located intracellular. The cloning showed that it was a double infection with *Enterococcus faecium *and an *Escherichia *like bacterium, but it was subsequently verified using the *in situ *hybridisation with a gamma proteobacteria probe that it was only the *Escherichia *like bacterium which infiltrated the superficial ulcerations and were found intracellular in epithelial cells and within neutrophilic granulocytes. Enterobacterial infection in the intestine is a common phenomenon, but it is rare to find these infections in the stomach and it has never before been reported in adult horses. This result is very intriguing but further studies need to clarify how common this phenomenon is in horses. Also, whether this type of infection is of primary or secondary origin would need further clarification. The *Escherichia *like clones all had 100% 16S rRNA gene similarity to both *E. fergusonii *and *Shigella flexneri*. Thus, in this study, it can not be precised experimentally which of these two organisms that were present in this glandular lesion. However, humans have been reported to be the only natural host for *Shigella *[17] whereas *E. fergusonii *has been associated with a wide variety of intestinal and extra-intestinal infections in both humans and animals including horses[18,19]. It is therefore most likely that the *Escherichia *like bacterium found in this study belongs to *E. fergusonii*. Studies have reported *E. fergusonii *as an emerging pathogen and associated with especially bacteraemia and wound infection but its precise role in infections in both humans and animals still has to be elucidated [20].

### Microbiology in the samples

The environment in the glandular stomach is generally very hostile toward microbes [21]. It is well established that, unlike humans and dogs that are meal feeders, horses are continuous acid producers, probably due to a continuous feeding pattern [22,23]. The pH in the ventral part of the equine stomach is stable at around pH 1-3 throughout the 24 hour period [24], consequently the relative low diversity of bacteria observed in mucosal samples in this study was not unexpected.

The characteristic morphological phenotype of large cocci growing in regular tetrads was established to be a clone with a 99% similarity to *Sarcina ventriculi*. This organism is known to be able to grow in stomach contents and has the characteristic tetrade structure when grown from pH 1- pH 3 [25]. In the current study, the finding of these organisms could not be established to be part of any specific pathology, as they were found in low numbers in the paired samples (i.e. lesion and normal), as well as in the control samples. Sarcina-like bacteria have been found in a variety of species, where they have been supposed to cause abomasal bloat, haemorrhage and ulcers in lambs and goat kids [26,27] and a possible link to gastric dilatation in both dogs and horses has also been suggested [28]. No evidence of gas accumulations was observed macroscopically in any of these horses and hence it does not seem that the presence of *Sarcina ventriculi *contributed to the pathology observed in these horses.

It was not surprising that Lactobacillus (*Lactobacillus salivarius*) was found in the studied tissues and it has previously been reported that several *Lactobacillus *spp., including *L. salivarius*, are present in healthy horses [16,29]. The proximal equine stomach functions as storage for feed, as well as a compartment for intragastric fermentation. The ecosystem in this region consists of both anaerobic and lactate-utilizing bacteria in large numbers, which are responsible for the increase in volatile fatty acids upon fermentation of carbohydrates [30]. Especially Lactobacilli were found adhering to the epithelium in the proximal part of the equine stomach [31] and these bacteria will likely pass to the glandular stomach as part of the normal turn-over. We have only examined subsamples and more bacterial taxa will be found in the healthy part of the glandular stomach if a more comprehensive microbiota community study was done.

### Validity of the findings of Helicobacter

None of the tissue samples from the antrum region demonstrated positive signals from the *Helicobacter spp*. probe in this study and no spiral shaped bacteria were noted using the FISH technique either. In a recent study from Venezuela, spiral shaped bacteria were reported in biopsies from the cardiac region of the equine stomach stained with the Warthin-Starry stain [12]. *Helicobacter spp*. known to be able to colonize the stomach produce large amounts of cytoplasmic urease[32] The rapid urease test used in this investigation, Pyloritek^®^, detects the urease activity of the tissue sample by the production of ammonia when urea is present. It is extensively used in human practice to detect gastritis caused by *Helicobacter spp*. The positive and negative predictive values were between 98.1-100% and 95.8-100%, respectively in a study testing human patients before and after eradication of the bacterium [33]. In this study, no positive tests were found, indicating that the biopsies in the present study contained no bacteria with the ability to produce urease.

## Conclusions

Gastric *Helicobacter *spp. was not found and could not be linked to the stomach lesions of the 36 horses analyzed in this study. The pathology found in this study included polypoid structures, hyperplastic rugae and small erosions, but bacterial involvement was found in only one case of an erosion. In this lesion, an Escherichia-like clone, most likely *E. fergusonii*, was found intracellular. Whether this was a primary or secondary infection could not be concluded. Very limited amounts of bacteria in general were found in the equine glandular region as expected. Thus, detection of a moderate to high amounts of any bacteria at the glandular mucosa level, as well as in the crypts should be cause for concern as this does not seem to be a normal finding in the equine glandular stomach. Further studies involving bacteria and the relation to gastric lesions of horses with confirmed clinical signs are warranted, as these horses were not included in the current study.

## Methods

### Horses and study design

The study was done as a cross-sectional study of stomachs from a population of 63 abattoir horses in Denmark. Horses were approved by the Veterinary Officer as healthy for slaughter. Horses were stunned with a captive bolt and exsanguinated. The stomach, including 5 - 10 cm of the distal esophagus and 10 cm of the proximal duodenum, was removed immediately after evisceration and opened along the greater curvature. Ingesta were removed and if necessary, the mucosa was gently rinsed with a minimum of tap water before inspection. Only stomachs with gross lesions in the glandular mucosa were included, as well as seven control stomachs with no gross evidence of gastric lesions.

**Figure 6:**
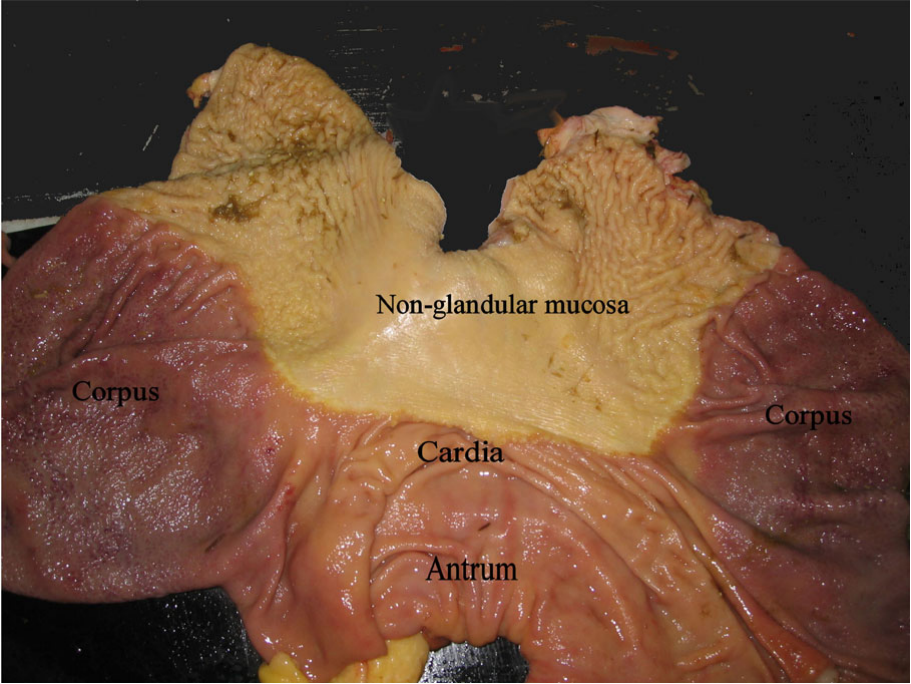
**Anatomical regions of the stomach opened along the greater curvature**. The non-glandular region has a white appearing epithelium, whereas the glandular region is shades of red. They are separated by the *Margo plicatus*. The three sampled regions include: Cardia as the small strip area just below and along the *margo plicatus*, the corpus region containing acid, pepsinogen and mucus secreting glands (dark red) and the antrum region containing primarly mucus and gastrin secreting glands.

Glandular lesions were defined as the mucosa having an abnormal macroscopic appearance i.e. hyperaemic, increased thickness, erosions or ulcers. The anatomical positions of the lesions were noted as: The cardia, corpus or antrum region (Fig. 6).

### Sampling procedure

From each stomach with glandular lesions, three tissue samples where obtained of the largest lesion (A, B, C) as well as three paired normal appearing tissue samples (a, b, c) from the same anatomical region, but at least at least 5 cm away. A/a: a small, biopsy size (0,5 × 0,5 cm) mucosa sample was obtained for immediate urease testing with the Pyloritek ^® ^assay according to the manufactures instructions. Tests were read after a 60 minute standard time and results noted as positive or negative. Samples B/b: a 3 × 3 cm full thickness tissue sample including mucosa and submucosa were obtained for FISH and fixed in 10% buffered formalin. After 24 hours fixation the samples were transferred to 70% ethanol, paraffin-embedded, sectioned at 3 μm and mounted on SuperFrost/plus slides (Menzel-Gläser, Braunschweig Germany).

Samples C/c: a third pair of tissue samples for cloning and sequencing was obtained and snap frozen using dry ice (If lesion size allowed it).

From the seven control stomachs with no macroscopic gastric lesions, samples a, b and c were taken from the normal appearing mucosa of the antrum. Three of these horses were additionally sampled in the cardia, corpus and duodenum as well.

The sampling procedures took place from August to October 2007. Historical data regarding previous health of the horses could not be obtained.

### Fluorescent In Situ Hybridisation for bacteria

For microbial detection, the tissue sections were hybridized simultaneously with two 16S rRNA probes labelled with different fluorophores. The oligonucleotide probe S-D-BACT-0338-a-A-18 targeting Bacteria (5'GCTGCCTCCCGTAGGAGT3') [34] was 5' labeled with the fluorescein isothiocyanate and with isothiocyanate derivative Cy3. The oligonucleotide probe HEL717 targeting the Helicobacter genus (5'AGGTCGCCTTCGCAATGAGTA3') [35] was 5' labeled with isothiocyanate derivative Cy3. To verify the cloning results a third and fourth probe, L-C-gProt-1027-a-A-17 (5'GCCTTCCCACATCGTTT3') targeting 23S rRNA of Gammaproteobacteria was 5' labeled with the fluorescein isothiocyanate and probe S-G-Enteroco-184 (5'CAAATCAAAACCATGCGG3') was Cy3 labeled targeting 16S rRNA of *Enterococcus *spp[36]. All probes were synthesised at DNA Technology, Aarhus, Denmark. The slides were deparaffinized in xylene and transferred to 100% alcohol for 30 min before hybridisation. The hybridisation was carried out at 45°C with 40 ml of hybridisation buffer (100 mM Tris [pH 7.2], 0.9 M NaCl, 0.1% sodium dodecyl sulfate) and 200 ng of each probe for 16 hours in a Sequenza Slide Rack (Thermo Shandon, Cheshire, UK). The samples were then washed three times in prewarmed (45°C) hybridisation buffer for 15 min and subsequently three times in prewarmed (45°C) washing solution (100 mM Tris [pH 7.2], 0.9 M NaCl). The samples were rinsed in water, air dried and mounted in Vectashield (Vector Laboratories Inc., Burlingame, CA, USA) for epifluorescence microscopy. An Axioimager M1 epifluorescence microscope equipped for epifluorescence with a 100-W HBO lamp and filter sets 43 and 38 were used to visualize Cy3 and fluorescein, respectively. Images were obtained using an AxioCam MRm version 3 FireWiremonocrome camera and the software AxioVision version 4.5 (Carl Zeiss, Oberkochen, Germany).

Evaluation of the epifluorescence microscopy was performed by description of the subjective amount, morphologic appearance and location of fluorescing cells apparent in each tissue sample. In addition, all tissue sections were stained by H&E and evaluated histopathologically.

### 16S rDNA amplification and cloning

After the detection of bacteria using FISH, sub samples from horses demonstrating bacteria of various morphologies were chosen for 16S rRNA gene cloning. The DNA was isolated from 4 tissue samples by using the Easy-DNA kit (Invitrogen, Tåstrup, Denmark) according to the manufacturer's instructions. The 16S rRNA gene was amplified using primers S-D-Bact-0008-a-S-20 (5'-AGAGTTTGATCMTGGCTCAG-3') [37] and S-*-Univ-1492-a-A-19 (5'-GGTTACCTTGTTACGACTT-3') [38]. PCR cycling consisted of an initial denaturation at 94°C for 6 min; followed by 30 cycles of denaturation at 94°C for 30 s, annealing at 55°C for 45 s and extension at 72°C for 2 min; and a final extension at 72°C for 3 min. Amplified DNA was verified by electrophoresis on agarose gels. The PCR products were purified using the QIAquick PCR purification kit columns (Qiagen GmbH, Hilden, Germany). To create blunt-ended DNA the following was mixed in a 0.5-ml microcentrifuge tube, 4 μl of 5 × T4 DNA polymerase buffer, 14.7 μl of purified PCR product 0.8 μl of dNTP (2.5 mmol l^-1 ^each) and 0.5 μl (1.2 U) of T4 DNA polymerase (Invitrogen) and incubated at 12°C for 15 min. The T4 DNA polymerase was heat-inactivated, and the blunt-ended DNA was purified using the QIAquick PCR purification kit columns (Qiagen GmbH) and eluted in a final volume of 10 μl of double-distilled water. Following the manufacturer's descriptions the cloning was performed by using a Zero blunt TOPO cloning kit (Invitrogen). Ten colonies from each cloning were picked and sequenced on an automatic sequence analyser (ABI PRISM 373 DNA Sequencer; PE Biosystems, Foster City, CA, USA) by using the two standard vector primers (T3 and T7) included in the kit. The sequence was assembled in Bionumerics version 4.0 (Applied Math, Sint-Martens-Latem, Belgium) and checked for chimeras both by blasting the individual sequences in GenBank http://www.ncbi.nlm.nih.gov and by the software Pintail version 1.1 http://www.cardiff.ac.uk/biosi/research/biosoft/. The phylogenetic analysis of the clones belonging to the *Escherichia *genus was done by downloading 16S rRNA gene sequences longer than 1,200 bp from the RDP v.9 database of the *Escherichia *type strains http://rdp.cme.msu.edu. The sequences were trimmed to the same length of 1327 bp and aligned pairwise (UPGMA) followed by a global sequence alignment. A final phylogenetic tree was constructed by using the WARD algorithm where *Enterobacter sakazakii *(AB004746) was used as outgroup.

## Authors' contributions

LH conceived and designed the study, collected and prepared the tissues, performed Fluorescence *In Situ *Hybridisation and drafted the manuscript. TKJ and LM assisted in designing the study, making microscopic images, performed the cloning and sequencing and drafted the manuscript. SNO participated in designing the study and helped draft the manuscript. All authors read and approved the final manuscript.
